# Silica/Proteoliposomal Nanocomposite as a Potential Platform for Ion Channel Studies

**DOI:** 10.3390/molecules27196658

**Published:** 2022-10-07

**Authors:** Rocío Esquembre, María Lourdes Renart, José Antonio Poveda, C. Reyes Mateo

**Affiliations:** Instituto de Investigación, Desarrollo e Innovación en Biotecnología Sanitaria de Elche (IDiBE), Universidad Miguel Hernández (UMH), 03202 Elche, Spain

**Keywords:** immobilization, ion channel, nanostructured materials, proteoliposomes, silica-matrices

## Abstract

The nanostructuration of solid matrices with lipid nanoparticles containing membrane proteins is a promising tool for the development of high-throughput screening devices. Here, sol-gel silica-derived nanocomposites loaded with liposome-reconstituted KcsA, a prokaryotic potassium channel, have been synthesized. The conformational and functional stability of these lipid nanoparticles before and after sol-gel immobilization have been characterized by using dynamic light scattering, and steady-state and time-resolved fluorescence spectroscopy methods. The lipid-reconstituted KcsA channel entrapped in the sol-gel matrix retained the conformational and stability changes induced by the presence of blocking or permeant cations in the buffer (associated with the conformation of the selectivity filter) or by a drop in the pH (associated with the opening of the activation gate of the protein). Hence, these results indicate that this novel device has the potential to be used as a screening platform to test new modulating drugs of potassium channels.

## 1. Introduction

The conventional sol-gel methodology that is used for the preparation of silica glasses is based on the hydrolysis of alkoxides such as tetramethylorthosilicate (TMOS) to yield porous and transparent 3D silica-based networks with high chemical, mechanical, thermal, and biological stability. In recent years, several modifications of these sol-gel systems have been described in order to improve their final properties [1,2]. Among them, the nanostructuring of the matrices through the inclusion of nanoparticles, such as carbon nanotubes, quantum dots, conjugated polymer nanoparticles, magnetic nanoparticles, or Au and Ag nanoparticles, allows both of the components to combine and synergize with each other, thereby resulting in structures with novel properties and functionalities. The potential applications of such materials include electronic circuits, sensors and biosensors, information storage, generators and transformers, or controlled release devices [3,4,5,6]. The crucial aspect in obtaining such nanocomposite materials is to prevent the aggregation or agglomeration of the nanoparticles, thus avoiding the loss/change of functionality.

Liposomes are spherical nanoparticles composed of phospholipids that self-assemble into at least one bilayer. They are used in a broad range of applications, including membrane models, drug and gene delivery, diagnostics, biosensor development, and cosmetic or food product manufacturing [7]. Despite their many applications, their use faces many obstacles, the most critical of which being their stability, which is strongly affected by factors such as temperature, pH, and ionic strength. Different strategies have been developed to overcome these difficulties. Particularly, the immobilization of liposomes in a silica matrix to give rise to nanostructured matrices is an interesting alternative that avoids their aggregation [8,9]. In addition, the nanostructured matrix acquires new properties and functionalities characteristic of liposomes, such as the ability to encapsulate hydrophilic and hydrophobic drugs and release them in a controlled manner [10].

Proteoliposome nanoparticles are liposomes with membrane proteins embedded in their lipid bilayer that present an additional level of complexity. The nanostructuration of the sol-gel matrices with the proteoliposomes brings additional applications to these materials, such as the development of platforms for the screening of potential modulators of their biological activity or biosensors where the protein is used as a recognition element. Nevertheless, there are only a few studies on these systems since they require the liposomes to retain their integrity during the encapsulation process [11]. 

In previous studies, our group had described the fabrication of liposome-doped silica nanocomposites using a sustainable methodology based on the sol-gel process, and we explored the effect of immobilization on the physical properties of the lipid membranes. In these works we reported that the electrostatic interactions between the phospholipid polar heads and the negatively charged silica surface of the porous matrix play a crucial role in preserving the fluidity and integrity of the immobilized bilayer [8,9]. Therefore, we concluded that the presence of the negatively charged phospholipids in the liposomes is highly recommended to minimize the interactions with the pore walls in systems where the structural integrity and fluidity of the bilayer must be preserved, such as in the reconstitution of membrane proteins. 

**Scheme 1:**
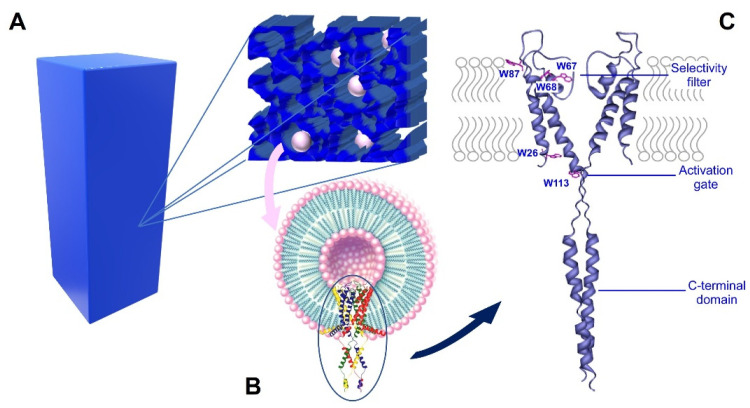
Nanostructured silica matrices with KcsA proteoliposomes. Panel (**A**) is a representation of the silica matrix that was obtained after gelation, containing KcsA@Lipo within its pores. Panel (**B**) shows the Kcsa@Lipo particles that are formed by KcsA tetramer that is reconstituted in liposomes, adopting an inside-out configuration (C-terminal domain exposed to the extravesicular space). Panel (**C**) shows the ribbon structure of KcsA, showing the five Trp residues that are mainly responsible of the intrinsic protein fluorescence, the C-terminal domain, the selectivity filter and the activation gate. Only two subunits are represented for clarity (PDB: 3EFF).

The present work describes the nanostructuring of sol-gel silica matrices with proteoliposomes containing the prokaryotic K^+^ channel KcsA. K^+^ channels are integral oligomeric membrane proteins that are involved in a multitude of physiological processes, such as the regulation of electrical activity, signal transduction, or the maintenance of the osmotic balance [12]. Particularly, KcsA is a pH-gated, voltage-modulated homotetrameric K^+^ channel that is cloned from the soil bacteria *S.lividans* whose high-resolution structure was solved in 1998 [13,14]. It is arranged around a central aqueous pore where the K^+^ cations specifically and rapidly permeate down to their electrochemical gradient [13]. Due to its homology to many eukaryotic K^+^ channels and its lower level of complexity, KcsA has been extensively used as a model to study the molecular basis of their functional properties. As any other K^+^ channels, KcsA is a highly dynamic entity that is able to respond to several stimuli and allosteric modulators through conformational changes in different protein domains. The most well-known conformational transitions occur at the selectivity filter (SF) and the activation gate (Scheme 1C). The selectivity filter is the portion of the pore that is mainly responsible for the selectivity, conduction, and inactivation properties of the protein, and its ability to adapt to the type and concentration of the cation that is present in the media [15,16]. The conformational plasticity of this domain is also allosterically associated with the conformation of the activation gate, which is at the opposite end of the channel. As part of the activation gate, we found the pH sensor, which is formed by a network of negatively and positively charged amino acids [17]. A decrease in the pH (<4.0) is sensed by these residues, thereby inducing a conformation change that opens the intracellular half of the KcsA and subsequently modifies the conformation of the SF from a conductive to an inactivated (non-conductive) form [18]. Interestingly, entering into this inactivated state is modulated by several factors, including the presence of anionic phospholipids that are bound to annular and/or non-annular binding sites at the protein surface [19]. The negatively charged phospholipids were also described as essential for the effective and proper folding of KcsA during the protein synthesis in the bacteria [20,21,22]. 

Several biophysical techniques have been used to characterize the conformational landscape of KcsA, including steady-state and time-resolved fluorescence spectroscopy. Particularly, the presence of five Trp residues per monomer (Scheme 1C) was exploited to characterize the changes on both the SF and the activation gate due to their privileged location in the transmembrane section [15,23,24]. Other techniques such as circular dichroism, although very suitable for detecting conformational changes, is not appropriate for these studies because the scattered light from the reconstituted vesicles interferes with the measurements [23].

In the present study, the proteoliposomes of KcsA (KcsA@lipo) were prepared with the wild-type protein reconstituted in lipid vesicles of different composition. The changes in the intrinsic protein fluorescence were used to monitor the success of the reconstitution as well as to evaluate the effects of the immobilization on the conformation and thermal stability of the protein. Specifically, the immobilization monitoring was carried out by observing the changes in the emission spectra and fluorescence lifetimes. The conformational response of the immobilized channel from the spectroscopic behavior of the protein at different pHs as well as in the presence of conducting and blocking ions, were used as indicators of the channel activity in the silica composite. In addition, the fluorescent probe Laurdan was used to analyze how immobilization affects the physical state of the lipid component of KcsA@lipo. The results presented in this work support the potential use of KcsA@lipo nanostructures immobilized in sol-gel matrices as a high throughput screening device to test new modulators of the ion channel activity.

## 2. Results and Discussion

### 2.1. Characterization of KcsA@lipo Nanoparticles 

The KcsA@lipo nanoparticles were characterized in a step that was prior to immobilization by recording the fluorescence spectrum of the protein, which is mainly from tryptophan residues, and by determining the size of the formed particles. Two kind of nanoparticles, KcsA@Aso and KcsA@PE:PG, were formed by the reconstitution of KcsA in lipid vesicles of different composition: asolectin or a DOPE:DOPG (7:3) mixture, respectively. Asolectin is a soybean extract whose composition may vary, but it has zwitterionic lipids predominantly and it has been shown to be suitable for reconstituting different ion channels while preserving their activity. The second of the systems, DOPE:DOPG, is commonly used to recreate prokaryotic membranes, and it has been specifically recommended to reconstitute KcsA [20,25].

**Figure 1:**
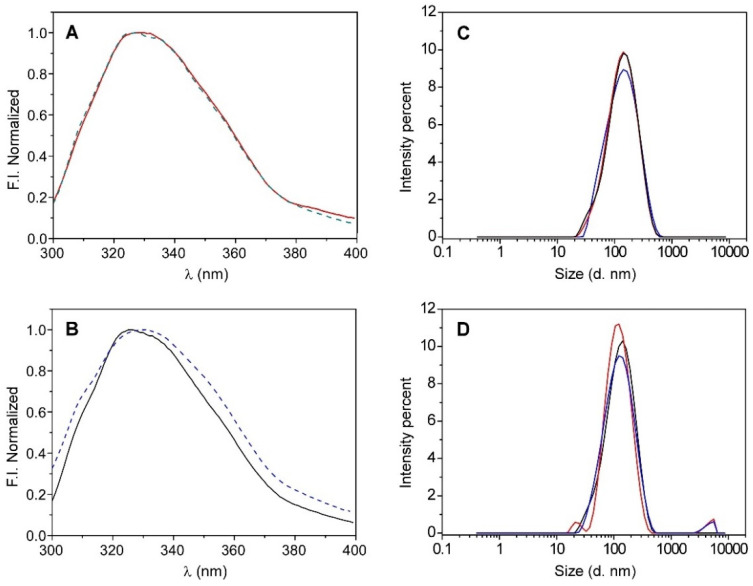
Emission spectra of KcsA (panel (**A**)) after reconstitution it in liposomes, KcsA@lipo, that are composed of asolectin (red line) or DOPE:DPPG (7:3) (green dashed line), and (panel (**B**)) it being in solution in a monomeric (blue dashed line) and a tetrameric (black line) state. DLS size distributions by intensity recorded in triplicate for KcsA@lipo (panel (**C**)) and KcsA@Aso (panel (**D**)) nanoparticles.

The results show that there are no significant differences in the emission spectrum of the protein regardless of the lipid composition that was used in the reconstitution (Figure 1A). Moreover, if these spectra are compared to those from the native KcsA tetramer, they are very similar, but different from the denatured KcsA monomer, which shows a pronounced red shift and broadening in the emission spectrum (Figure 1A,B). These spectral differences between the monomeric and tetrameric KcsA conformations have been previously reported, and they are used to monitor the dissociation of the protein [26,27]. This result suggests that both of the lipid systems (KcsA@Aso and KcsA@PE:PG nanoparticles) allow the channel to adopt its native tetrameric conformation.

The size of the formed nanoparticles was determined by dynamic light scattering (DLS), which obtained hydrodynamic diameter values that were quite similar and slightly higher than 100 nm in both of the lipid systems (see Table 1 and Figure 1C,D), thereby suggesting that the type of lipid that was used to reconstitute the channel does not seem to significantly influence the nanostructure formation. However, while the particle size distribution of the KcsA@PE:PG system was monomodal (Figure 1C), a second population with larger diameters was often identified when the KcsA was reconstituted in asolectin, which could indicate a slight tendency of the KcsA@Aso nanoparticles to aggregate (Figure 1D and Table 1). Taking this into account, together with the variability between the different asolectin extracts and the possible presence of traces of protein contaminants, it was decided to perform the rest of the tests in this work with the KcsA@PE:PG system, whose lipidic composition is well defined. 

### 2.2. Immobilization and Stability of the KcsA@PE:PG Nanoparticles in the Sol-Gel Matrix

To determine the correct immobilization and stability of the KcsA@PE:PG nanoparticles in the sol-gel matrix (Scheme 1), several fluorescence studies were carried out. The intrinsic fluorescence of the protein was used to evaluate changes in its conformation, long-term and temperature stability, as well as its solvent accessibility. The effect of the immobilization on the lipid component of these nanoparticles was also assessed by using the external fluorescent probe Laurdan. 

#### 2.2.1. Conformation Plasticity of the Selectivity Filter Is Preserved after the Immobilization 

As previously described, the SF is the domain that is mainly responsible for the functional properties of the ion channels. Its conformation can be modulated by the type and concentration of blocking or permeant cations, among other factors. In KcsA, the intrinsic fluorescence emission spectrum is sensitive to the SF conformation, since two of the five Trp residues per monomer, Trp67 and Trp68, are located at the pore-helix, a structure that is linked to the filter through several H-bond interactions [24,28]. Here, the changes of this spectrum were used to test if the conformational plasticity of the SF of the KcsA@PE:PG nanostructures was maintained after the immobilization in the nanocomposite silica matrix. Such changes in the KcsA fluorescence spectrum, although they were quite small, are highly reproducible and have been used in previous works not only to detect SF conformational changes but also to determine K^+^ and Na^+^ dissociation constants [23,24]. 

**Figure 2:**
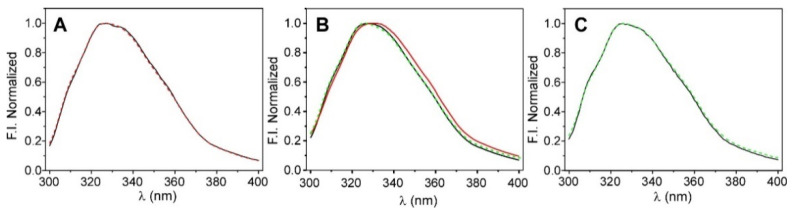
Steady-state fluorescence characterization of KcsA@PE:PG nanostructures. Panel (**A**): emission spectra of the free-in-solution (black line) and sol-gel immobilized (red dashed line) nanoparticles measured in 20 mM Hepes, 100 mM KCl, and pH 7.0 buffer. Panel (**B**): shift on the emission spectra when the initial condition (100 mM K^+^, black line) was changed to 200 mM Na^+^ (red line) and then reverted to 100 mM K^+^ (green dashed line). Panel (**C**): comparation of the intrinsic fluorescence emission spectra of the KcsA@PE:PG nanostructures in the nanocomposite that was stored at +4 °C from day 1 (black line) to day 15 (dashed green line).

As illustrated in Figure 2A, the fluorescence spectra of the KcsA@PE:PG nanoparticles (100 mM K^+^, pH 7.0) in the solution and in the nanocomposite silica matrix were identical, thus suggesting that the incorporation of the protein into the silica matrix does not affect the overall tertiary structure of the protein. In order to explore the long-term stability of the KcsA@PE:PG nanostructures that were immobilized in the sol-gel matrix, some of the samples were kept at +4 °C, and then the protein emission spectrum was collected. The comparison of the day 1 and day 15 spectra shows that there were no detectable changes (Figure 2C), thus suggesting that the channel structure remains intact and stable after this period in the silica matrix.

Afterwards, we proceeded to test if the conformational plasticity of the SF was maintained. To do so, a monolith containing the KcsA@PE:PG nanostructures in 20 mM Hepes, 100 mM KCl, and pH 7.0 buffer were extensively washed with a buffer to replace the permeant cation K^+^ with Na^+^, the physiological relevant blocker. Under this experimental condition, the SF conformation shifted from a conductive conformation to a collapsed-nonconducting form. This shift in the equilibrium is characterized by a small movement of the fluorescence emission spectrum to higher wavelengths [23], which was in fact detected in the immobilized particles (Figure 2B). Moreover, this process was fully reversible since the samples in the Na^+^ returned to the K^+^ behavior after washing again the monolith in K^+^ buffer. The behavior, starting with the monoliths in sodium as the initial condition, was analogous: the starting spectrum red-shifted due to the presence of Na^+^, then blue-shifted by adding K^+^, and returning to the initial spectrum when reverting to Na^+^ (data not shown). These results suggest that the conformational equilibrium of the SF is preserved after the immobilization of the KcsA@PE:PG nanostructures into the sol-gel matrices, even though the spectral shift is small. To confirm these observations, we decided to also test the thermal stability of KcsA. 

**Figure 3:**
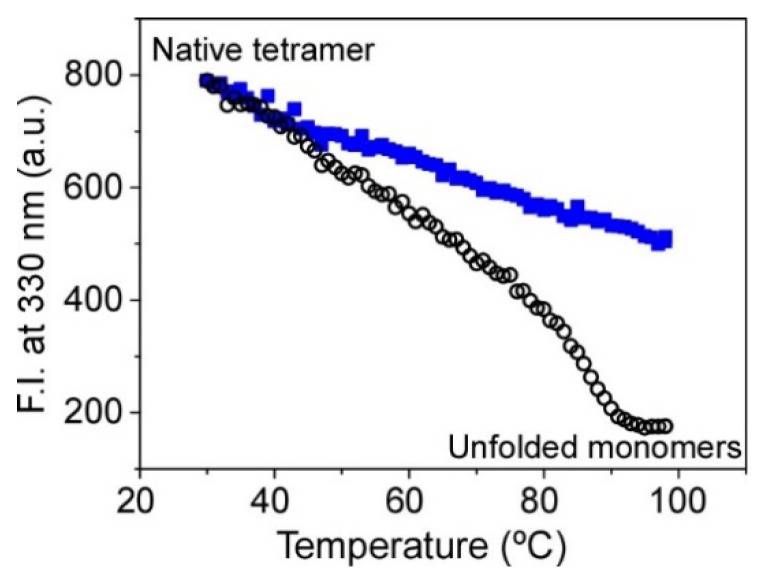
Changes on the thermal stability of KcsA@PE:PG nanostructures immobilized in the sol-gel monoliths associated with the ion occupancy of the SF. The midpoint temperature of the thermally induced protein denaturation process (*t_m_*) was 90 ± 1 °C (*n* = 2) in 200 mM Na^+^ (empty black circles) and >99 °C in 100 mM K^+^ (full blue squares).

The thermal denaturation of the KcsA channel has been widely used to test the stability of the protein in different experimental conditions. This thermotropic behavior is sensitive to the type and concentration of the cations within the selectivity filter, the drop of the experimental pH, or the binding of phospholipids [27,28]. As illustrated in Figure 3, the dissociation of the native tetrameric channel to the partial unfolded monomers exhibits a sigmoidal behavior. As it was previously reported in detergent-solubilized protein [28], the *t_m_* of the process in the KcsA@PE:PG nanostructures that were encapsulated in the sol-gel monoliths is still sensitive to the cations present in the sample buffer, i.e., the channel had a lower thermal stability in the presence of the blocking cation Na^+^ (*t_m_* = 90 ± 1 °C, *n* = 2) than in K^+^ (permeant cation). In fact, the stability of the K^+^•KcsA complex was so high that we were not able to calculate the *t_m_* parameter (>99 °C) under this experimental condition. This is in agreement with the previous Fourier transform-infrared (FT-IR) experiments on the asolectin-reconstituted WT KcsA channel in 100 mM K^+^ where a *t_m_* of ~117 °C was determined [23,29]. 

Interestingly, performing a thermal denaturation assay on a reconstituted sample of KcsA that is immobilized in a sol-gel matrix offers a great advantage compared to the analysis of the proteoliposomes in a solution: the denaturation curves are highly reproducible since the aggregation process during the heating of the sample is avoided, thereby preventing the appearance of artefacts that could affect the determination of the *t_m_* parameter.

#### 2.2.2. The KcsA@PE:PG Nanoparticles Present a Correct Gating Behavior in the Sol-Gel Matrix 

**Figure 4:**
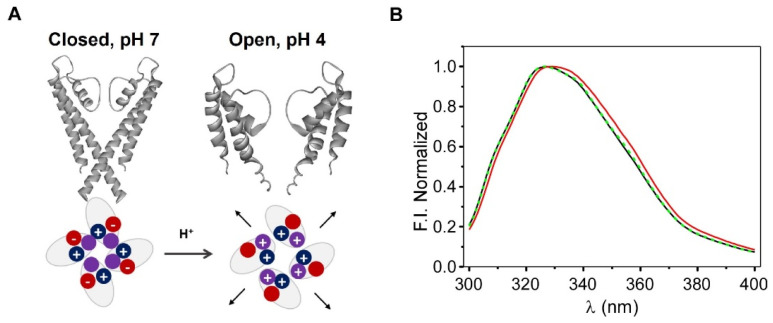
Panel (**A**): Scheme of the opening of the KcsA channel and the pH sensor, showing the charge of the aminoacids that were involved in channel opening at the activation gate. Panel (**B**): Changes in the fluorescence emission spectra of the KcsA@PE:PG nanostructures that were immobilized in the sol-gel matrices, which were associated with this gating process; pH conditions: pH 7 (black line), pH 4 (red line), and reversion to pH 7 (dashed green line).

As stated in the Section 1, KcsA is a pH-gated channel. The pH sensor is located at the activation gate, in the intracellular interface of the transmembrane section of the protein (Scheme 1 and Figure 4A). A decrease in the experimental pH from neutral to acid (<4.0) changes the interaction network of this domain and induces the opening of the intracellular half of the channel [17]. This is the first step for the conduction process to take place. Even though the typical functional characterization of the K^+^ currents is performed by electrophysiological techniques, these approaches cannot be applied in the immobilized silica matrices. Nevertheless, as a preliminary step to explore whether the channel remained functional after immobilization, we investigated whether the intrinsic fluorescence emission spectra of KcsA suffered a red shift when the pH was exchanged from 7.0 (closed state) to 4.0 (open state), as it was described in the detergent-solubilized channel [24]. In this case, the changes in the fluorescent properties should arise from the intracellular Trp 26 and Trp113 residues, which are located near the activation gate, and from the residues that are near the SF (Trp 67 and 68). Figure 4 illustrates the spectra that were obtained for a monolith that was prepared at pH 7.0 (20 mM Hepes, 100 mM KCl), after washing it for 24 h with pH 4.0 buffer (10 mM citric acid, 100 mM KCl) and reverting it to pH 7.0. Since the predicted shifts of the emission were detected, we suggest that the conformational equilibrium of the inner gate is preserved after the immobilization and that the immobilized channels should thus retain their native functional properties.

In summary, the modifications that were detected in the spectra of the immobilized protein, both by cation type and pH, and the ability to reverse these modifications seem to confirm that the channel encapsulated in silica matrices is still capable of undergoing most of the conformational changes that are necessary for the permeation process to take place. 

#### 2.2.3. Time-Resolved and Fluorescence Quenching Studies Revealed Only Subtle Conformational Changes in the Overall Structure of Immobilized KcsA@PE:PG Nanoparticules 

**Figure 5:**
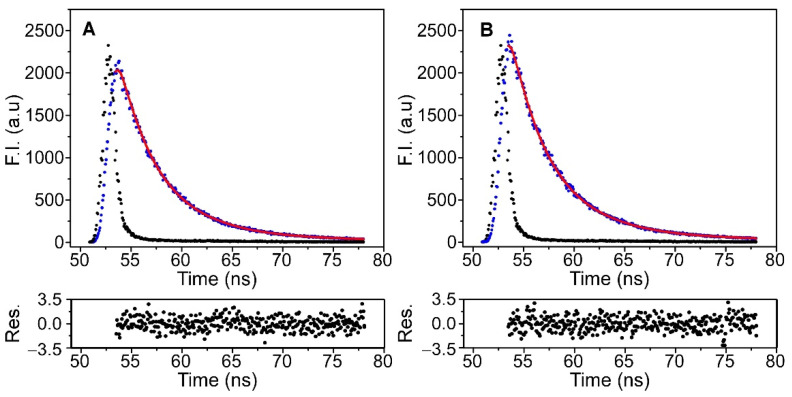
Time-resolved fluorescence decays of KcsA in the KcsA@PE:PG nanostructures in solution (panel (**A**)) and after their immobilization in silica matrices (panel (**B**)), which were collected at 25 °C (black dots). The instrumental function (blue dots), the fit to a biexponential equation (red line) and the residuals obtained for the fit are also shown.

To examine in more detail the effect of immobilization on the channel properties, time-resolved fluorescence experiments were carried out on the KcsA@PE:PG nanostructures before and after the immobilization process. This technique is more sensitive than the steady-state fluorescence characterization for detecting the existence of any alteration in the surroundings of the Trp residues. Nevertheless, the interpretation and concrete assignment of the different lifetimes in multi-tryptophan proteins, such as KcsA, is complex, and the experimental results are usually analyzed on the basis of the average lifetimes, <τ>. In the present study, the time-resolved fluorescence emission decay of the protein was satisfactorily fitted to a biexponential equation, both in the free KcsA@PE:PG nanoparticles and in the silica nanocomposite (Figure 5). The fitting parameters that were extracted from these analyses are listed in Table 2.

The results show that the average lifetime of the KcsA@PE:PG nanostructures slightly decreased after the immobilization. This shortening in the <τ> may be attributed to the occurrence of some kind of quenching process in the encapsulated sample. It is known that the fluorescence of Trp in solution is quenched in the presence of acidic groups, through proton or electron transfer mechanisms. The silica matrix has −OH and −O^−^ groups on its pore surface that could potentially quench the fluorescence of the Trp of KcsA. However, this is unlikely to happen given the location of these residues inside the channel pore, some of them anchored in the membrane. Furthermore, several studies show that the KcsA channel inserts into these type of liposomes in an “inside out” orientation, i.e., exposing its intracellular C-terminal domain (CTD) to the extravesicular milieu [23,30], therefore exposed it to the matrix pore. In any case, the presence of the CTD should minimize the possible contact between the pore surface and the annular tryptophan residues that are anchored on that side of the bilayer (Trp 16 and 113). Thus, a plausible explanation for the small decrease in <τ> is that the interaction of the CTD with the matrix wall could induce a small conformational change around one or more Trp amino acids, thereby leading to an effective quenching by some acidic side chains of the protein.

**Figure 6:**
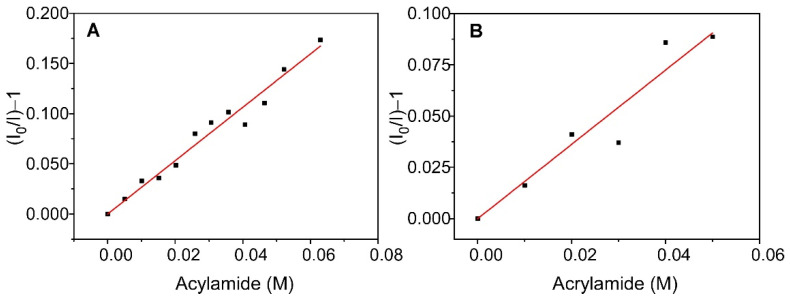
Stern–Volmer fits for the quenching by acrylamide of the fluorescence of KcsA@PE:PG nanostructures suspended in solution (panel (**A**)) and encapsulated in a sol-gel matrix (panel (**B**)).

In combination to the time-resolved characterization experiments, steady-state fluorescence quenching experiments were carried out to obtain additional information on the effect of immobilization on the KcsA properties. The quencher selected for this purpose was acrylamide, which is water soluble and practically does not penetrate the lipid bilayer. Therefore, this experiment provides information on the degree of water exposition of the channel Trp residues, and whether there is any difference between the free and immobilized systems. The addition of this quencher to the different samples produced a decrease in fluorescence intensity, although the effect was less pronounced in the case of the immobilized system. The obtained results are shown in Figure 6 and are represented as Stern–Volmer plots. The plots are linear over the range of the acrylamide concentrations that were studied, and they were satisfactorily fitted to the Stern–Volmer equation (Equation (3)).

From these fits, the value of K_SV,ef_ was obtained for KcsA@PE:PG in solution and in the sol-gel matrix, which together with the <τ> values that are displayed in Table 2, were used to calculate (Equation (4)), the bimolecular quenching constant of the process, k_Q,ef_ (Table 3). Note that these constants are apparent since it is a multi-tryptophan protein. The results show that, although the value of K_SV,ef_ is clearly higher for the non-immobilized system, the values of k_Q,ef_ are much closer, being only slightly lower for the immobilized KcsA. This behavior suggests that the accessibility of the quencher to the fluorescent residues of the protein is virtually unchanged by encapsulation, with only a very slight decrease in solvent exposure occurring after immobilization. However, it should be noted that the k_Q,ef_ is quite low even in solution, indicating that the fluorescent residues of the protein have poor accessibility to the aqueous medium.

#### 2.2.4. The KcsA Incorporation on DOPE:DOPG Liposomes and Immobilization Decreases Water Hydration/Mobility in the Lipid Bilayer

To investigate how immobilization affects the physical state of the KcsA@PE:PG lipid system, the behavior of the fluorescent probe Laurdan once incorporated into the membrane was explored. Laurdan is a polarity-sensitive fluorescent probe that is located at the level of the glycidic backbone in the lipid bilayer, and its fluorescence spectrum is strongly dependent on the lipid packing [31,32,33,34]. 

**Figure 7:**
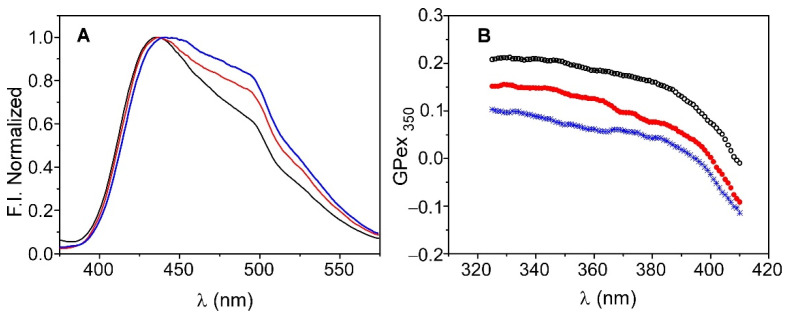
Emission spectra (panel (**A**)) and Generalized Polarization (GPex) (panel (**B**)) of Laurdan in liposomes of DOPE:DOPG (7:3) in solution (blue) and in KcsA@PE:PG before (red) and after immobilization (black).

Figure 7A shows the emission spectra of Laurdan in an aqueous suspension of DOPE:DOPG (7:3) in the absence of the channel, as well as in KcsA@PE:PG before and after immobilization. The three spectra display the two main bands that are characteristic of Laurdan; one is centered on 440 nm and the other is at approximately 490 nm, although their relative intensity is not preserved. It has been reported that the decrease in the intensity of the peak at 490 nm, relative to that at 440 nm, is caused by the decrease in water molecules in the bilayer and/or their lower mobility, which usually occurs in ordered phases due to increased lipid packing [31,32,34]. In this context, the results suggest that the incorporation of KcsA into the membrane, prior to its immobilization, already produces an alteration of the lipid bilayer, thereby increasing the packing and/or decreasing the quantity or mobility of the water molecules. The subsequent immobilization of KcsA@PE:PG in the silica matrices further enhances this effect. Such behavior is corroborated by the Laurdan GPex values that were obtained in the three systems using Equation (1) (Figure 7B). The decrease in the dynamics of the water molecules in the bilayer is reflected as an increase in the value of GPex. Thus, the highest GPex values correspond to the system formed by the immobilized KcsA@PE:PG, while the lowest values are found for the liposomes in suspension, which present typical values of lipids in a fluid phase. These results could be explained considering the tendency of zwiterionic lipids to interact with the walls of the silica matrix pores, as described in previous works by our group [8,9]. Such an interaction causes the lipid system to behave as if it were in a pure fluid phase but remaining in a somewhat more rigid state. In the case of the KcsA@PE:PG proteoliposomes, there is a 70% zwitterionic lipid component that probably interacted with the sol-gel matrix, this giving rise to the observed effect. 

In any case, this slight alteration of the physical properties of the lipid bilayer after the immobilization does not seem to have any effect on the conformation and function of KcsA and, therefore, on the potential application of the device as a screening platform.

## 3. Conclusions

The incorporation of liposome-based nanoparticles containing the potassium channel KcsA into silica matrices provides nanocomposites where these nanoparticles are correctly immobilized. The studies carried out by following the photophysical properties of the protein, show that the conformation, thermal, and long-term stability of the KcsA channel are retained once it is incorporate into the nanostructured material. Slight modifications of the lipidic bilayer hydration/packing were revealed by the Laurdan fluorescent probe, as a consequence of the KcsA presence into the bilayer and subsequent silica matrix entrapment. Nevertheless, these effects do not significantly impact the regular conformational changes on the selectivity filter (from conductive to blocked or inactivated forms) and opening of the activation gate of the channel. Thus, the activity of the channel is expected to be maintained in the nanocomposite, thereby revealing the potential usefulness of these materials as platforms for channel effector drug screening devices using similar silica/proteoliposomal nanocomposites, but with physiologically more relevant channels.

## 4. Materials and Methods

### 4.1. Reagents

The matrix precursor, tetramethyl orthosilicate (TMOS), and the phospholipids that were used, soybean asolectin, 1,2-Dioleoyl-sn-Glycero-3-Phosphoethanolamine (DOPE), and 1,2-Dioleoyl-sn-Glycero-3-[phospho-rac-glycerol] (DOPG), were obtained from Sigma-Aldrich Chemical Co. (Milwaukee, WI, USA). Spectroscopic grade acrylamide and analytical grade reagents that were used in the preparation of the buffer solutions (Hepes-Ac, citric acid, NaCl, and KCl) were also supplied by Sigma-Aldrich. The fluorescent probe 2-dimethylamino-6-lauroylnaphthalene (Laurdan) was purchased from Molecular Probes (Eugene, OR, USA), and it was used directly without any prior purification. The solvents that were used in the dissolution of lipids and probes (ethanol and chloroform) were from Merck, and the spectroscopic grade, ultrapure water was obtained by distillation and deionization using Milli-Q equipment (Millipore, Madrid, Spain).

### 4.2. Expression and Purification of KcsA

The wild-type KcsA channel (KcsA wt) was expressed and purified as previously described [26]. Briefly, the expression of the N-terminal hexahistidine-tagged protein was carried out in Escherichia coli M15 (pRep4) cells, and its purification was performed by immobilized metal affinity chromatography on a Ni^2+^-Sepharose 6 fast flow resin (GE Healthcare). The protein stock was stored at +4 °C in 20 mM Hepes, 5 mM n-dodecyl β-D-maltoside (DDM; Calbiochem), 100 mM NaCl, and pH 7.0 buffer. After purification, the protein concentration was determined by Bio-rad DC assay, relative to a bovine serum albumin standard.

### 4.3. KcsA Oligomeric State Characterization

The oligomeric state of KcsA that was reconstituted in proteoliposomes was checked by comparing the protein fluorescence spectra with the ones of the solubilized samples containing mostly tetrameric or monomeric species. The tetrameric and monomeric enriched samples were prepared by subjecting freshly purified KcsA wt samples (20 mM Hepes pH 7.0, 100 mM KCl, and 5 mM DDM) in a discontinuous sucrose gradient in the same buffer. Briefly, 2.1 mL for each sucrose fraction (20%, 15%, 10%, 10%, and 5%) and 0.2 mL of sample with KcsA (1.36 mg/mL) were subjected to ultracentrifugation (Beckman; 35,000 rpm, 16h, 4 °C, and SW41 rotor without brake), and the tubes were punctured to collect the fractions. SDS-PAGE was performed on the different collected aliquots and the ones with the highest percentage of monomer and tetramer were selected and subjected to a dialysis step to remove the sucrose. 

### 4.4. Liposome Preparation and KcsA Reconstitution

The detergent-solubilized protein was reconstituted in asolectin or in a mixture of DOPE:DOPG (7:3) liposomes using a 500:1 lipid:protein molar ratio. For this purpose, the appropriate volume of a lipid stock in chloroform (Asolectin or DOPE:DOPG 7:3) was pipetted into a glass vial and dried by evaporation under a gaseous stream of nitrogen, and it was then under vacuum for 3 h. Subsequently, the lipid was resuspended (by shaking and sonication) in the same buffer as the protein and both solutions were mixed by gentle agitation for 2 h. The samples were then diluted 10-fold, subjected to dialysis using the same buffer without DDM (20 mM Hepes, pH 7.0, 200 mM KCl or NaCl), and then centrifuged (45,000 rpm, T-45 rotor, 60 min, and 4 °C) and resuspended in the corresponding buffer. The same procedure was performed in parallel using only the lipid suspension to obtain liposomes without protein. The reconstituted protein samples (KcsA@Aso and KcsA@PE:PG) or protein-free liposomes were aliquoted and stored at −80 °C until use. 

### 4.5. Dynamic Light Scattering (DLS) Measurements

Proteoliposomes size determination was carried out by Dynamic Light Scattering (DLS) technique using a Malvern Zetasizer Nano-ZS instrument (Worcestershire, UK) with a Helium-Neon laser (λ = 633 nm). Measurements were performed at 25 °C in triplicate at an angle of 173° and using disposable cuvettes.

### 4.6. Labeling of Liposomes and Proteoliposomes

Incorporation of the Laurdan fluorescent probe into liposome membranes was performed by directly adding a few microliters of the stock solution of the probe in ethanol to the liposomes/proteoliposomes suspension up to a lipid/probe molar ratio of 1:500. The final concentration of organic solvent (ethanol) was always less than 2%.

### 4.7. Immobilization in Sol-Gel Monoliths

DOPE:DOPG liposomes and KcsA@PE:PG nanoparticles were encapsulated in silica matrices following the alcohol-free protocol previously described in [35,36]. For this purpose, a silica stock solution was prepared by mixing 5.88 mL of TMOS, 2.88 mL of H_2_O, and 0.06 mL of HCl (0.62 M), and subjected to constant shaking at 4 °C in a sealed vial. After 50 min, equal volumes of the resulting stock solution were mixed with distilled water and the mixture was subjected to rotaevaporation to a mass loss of 0.56 g per milliliter of stock solution that was added (the mass of alcohol resulting from hydrolysis of the alkoxide). Then, equal volumes of the rotaevaporated aqueous solution and the liposomes/proteoliposomes suspension in buffer were mixed in a small volume polymethylmethacrylate cuvette (10 mm × 5 mm) using a plastic separator. Gelation occurs rapidly after mixing the solutions. The obtained monolithic matrices (5 mm × 5 mm × 17 mm) were kept in 20 mM Hepes and with pH 7.0 buffer at 4 °C until use.

### 4.8. Steady-State Fluorescence Measurements

Fluorescence spectra measurements of KcsA and Laurdan were carried out using a SLM-8000C fluorimeter (SLM Instrument Inc., Urbana, IL, USA). Emission spectra were performed at 25 °C by using an excitation wavelength of 280 nm for the KcsA@PE:PG nanoparticles and 350 nm for Laurdan-containing samples. Changes in the Laurdan emission spectra were quantified from the calculation of the generalized polarization GPex) over a range of excitation wavelengths between 325 and 410 nm and collecting the emission at 440 and 490 nm [31,32].

(1) $$\mathrm{GPex}=\frac{I_{440\mathrm{nm}}-I_{490\mathrm{nm}}}{I_{440\mathrm{nm}}+I_{490\mathrm{nm}}}$$

The samples (immobilized or in suspension) were measured in 5 × 5 mm quartz cuvettes. Background intensities from the sol-gel matrix and/or liposomes were subtracted from the sample signal.

### 4.9. Time-Resolved Fluorescence Measurements

Total fluorescence intensity decays of KcsA@PE:PG samples were collected at 25 °C using a PTI model C-720 fluorescence lifetime instrument (Photon Technology International Inc., Laurenceville, NJ) with a stroboscopic detection system. The system uses a PTI GL-330 pulsed nitrogen laser pumping a PTI-GL-302 high-resolution laser dye. From the laser dye emission, the 590 nm wavelength was selected which was frequency doubled to 295 nm using a GL-303 frequency doubler coupled to an MP-1 sample compartment via an optical fiber. Emission was collected using magic angle conditions (Glan-Thomson polarizers) using a M-1010 emission monochromator and a stroboscopic detector equipped with a Hamamatsu 1527 photomultiplier. The data were analyzed with Felix 32 software applying a biexponential decay model and the average fluorescence lifetime was calculated according to [37]:

(2) $$\langle \tau \rangle =\frac{\sum_{i}\alpha_{i}\tau_{i}^{2}}{\sum_{i}\alpha_{i}\tau_{i}}$$

where α_i_ and τ_i_ are the amplitudes and the lifetimes, respectively, of individual components. 

### 4.10. Thermal Stability

Thermal denaturation of KcsA channel in the immobilized KcsA@PE:PG was performed using a Varian Cary Eclipse spectrofluorometer (Agilent) by monitoring the temperature dependence of the protein intrinsic fluorescence emission at 340 nm after excitation at 280 nm as in [27]. The midpoint temperature of the thermally induced protein denaturation process (*t_m_*) was calculated using a two-state unfolding model as in [27]. Samples were prepared in 20 mM Hepes with pH 7.0 buffer supplemented with 100 mM KCl or 200 mM NaCl.

### 4.11. Fluorescence Quenching Experiments

The fluorescence intensity of the Trp residues from the KcsA@PE:PG nanostructures was collected at 330 nm (λ_ex_ = 295 nm) in the presence of increasing amounts of acrylamide. For studies in sol-gel matrices, monoliths containing the same concentration of KcsA (0.6 μM, monomer based) were incubated separately with the acrylamide solutions for 48 h. Corrections due to inner filter effects [38] and sample dilutions were performed when necessary. The absorbances of the samples were measured using a Shimadzu spectrophotometer (UV-1603, Tokyo, Japan). The data were analyzed according to the Stern–Volmer equation (Equation (3)) and an effective Stern–Volmer constant, K_SV,ef_, was obtained directly from the slope of the fit [39].

(3) $$\frac{I_{o}}{I}=1+K_{\mathrm{SV},\mathrm{ef}}\left[Q\right]$$

where *I_o_* and *I* are the steady-state fluorescence intensities in the absence and presence of quencher, respectively, and [Q] is the concentration of quencher.

The bimolecular constant of the quenching process, k_Qef_, was calculated from the expression:

(4) $$K_{\mathrm{SV},\mathrm{ef}}=k_{\mathrm{Qef}}<\tau >$$

## Figures and Tables

**Table 1:** Parameters recovered from the DLS measurements: intensity weighted mean hydrodynamic diameter (Z-Ave), polydispersity index (PDI), and peak sizes of the distribution analysis by intensity indicating its relative area%.

Sample	Z-Ave (d.nm)	PDI	Peak 1 Mean Int d.nm (Area%)	Peak 2 Mean Int d.nm (Area%)	Peak 3 Mean Int d.nm (Area%)
KcsA@PE:PG	115.2	0.252	157.3 (100%)		
	115.6	0.238	154.2 (100%)		
	114.6	0.244	155.7 (100%)		
	* 115.1 ± 0.5	* 0.245 ± 0.007			
KcsA@Aso	109.0	0.242	143.3 (100%)		
	108.2	0.238	132.2 (95.9%)	4692 (2.2%)	22.9 (1.9%)
	108.8	0.252	139.5 (98%)	4590 (2%)	
	* 108.7 ± 0.4	* 0.244 ± 0.007			

* Mean value of the 3 measurements and standard deviation.

**Table 2:** Analysis of the fluorescence intensity decays obtained at 25 °C for the KcsA@PE:PG nanostructures in solution and immobilized in the sol-gel matrices.

Sample	α_1_	α_2_	τ_1_ (ns)	τ_2_ (ns)	<τ> ^1^ (ns)	χ^2^
KcsA@PE:PG solution	0.42	0.58	2.71	5.36	4.66	0.83
KcsA@PE:PG sol-gel	0.19	0.81	1.12	4.31	4.13	0.93

^1^ <τ> is the intensity-weighted lifetimes calculated according to Equation (2).

**Table 3:** Acrylamide fluorescence quenching constants (K_SV,ef_ and k_Q,ef_) of KcsA in KcsA@PE:PG nanostructures suspended in solution or immobilized in the sol-gel matrices.

Sample	K_SV,ef_ (M^−1^)	k_Q,ef_ (M^−1^s^−1^)
KcsA@PE:PG, solution	2.66	0.57
KcsA@PE:PG, sol-gel	1.81	0.44

## Data Availability

Not applicable.
